# Phytochemical screening and insecticidal activities of some medicinal plants against the maize weevil, *Sitophilus zeamais* (Motschulsky) (Coleoptera: Curculionidae)

**DOI:** 10.1038/s41598-024-59207-z

**Published:** 2024-04-15

**Authors:** Adugna Gindaba, Mulugeta Negeri, Bulti Abdisa, Reda Nemo, Chala Kitila

**Affiliations:** 1 Department of Biology, College of Natural and Computational Sciences, Dambi Dollo University, P.O. Box. 260, Dambi Dollo, Ethiopia; 2 Department of Plant Science, College of Agriculture and Veterinary Sciences, Ambo University, P.O. Box. 19, Ambo, Ethiopia; 3 Department of Chemistry, College of Natural and Computational Science, Ambo University, Ambo, Ethiopia; 4 Department of Plant Sciences, College of Agriculture and Natural Resource, Dambi Dollo University, P.O. Box. 260, Dambi Dollo, Ethiopia

**Keywords:** Phytochemical study, Maize weevil, Seed germination, Crude extract, Extraction solvents, Plant sciences, Zoology

## Abstract

This study emphasizes the phytochemical study of some locally available botanicals against maize weevils. Nine plant parts were collected from six plant species. The test plant powder (200 g) was suspended sequentially in 600 ml of petroleum ether, chloroform, acetone, methanol, and distilled water for 72 h with frequent agitation. Different concentrations of the crude extracts were applied to maize seeds at rates of 10 ml, 15 ml and 20 ml per 100 g. All treatments with different extracts at different rates of application showed significant differences (p < 0.05) in the cumulative mean percentage mortality of the maize weevil. The seed extract of *Maesa lanceolata* and *Croton macrostachyus* and the leaf extract of *Clausena anisata* showed cumulative percent mortality ranged 95.32–98.02% in 28 days after treatment application. There was no significant difference (p > 0.05) among all treatments for the prevention of F1 progeny emergence. In all extracts, *Clausena anisata* showed 100% inhibition of F1 progeny emergence. All treatments significantly reduced seed weight loss and damage. The treated maize seeds were germinated with an acceptable germination quality. In conclusion, an increased dosage of the extract resulted in significant mortality in maize weevils. The seed extracts of *Maesa lanceolata* and *Croton macrostachyus* and *Clausena anisata* leaf extract were observed to be the most promising botanical in protecting stored maize against maize weevil.

## Introduction

Agriculture is the backbone of the economy in most sub-Saharan African countries, contributing significantly to the Gross Domestic Product^1^. In agricultural sector, grains are the major product, of which maize is the main contributor. Globally, Maize (*Zea mays* L.) ranks third innext to wheat and rice in cereal production, making it an important crop for food security that helps to increase per capita energy consumption and income, particularly in developing countries^2,3^. Meeting the food demand of the rapidly increasing global population is emerging as a major challenge to humankind. The population is expected to grow to 9.1 billion people by 2050, and approximately 70% extra food production will be required to feed them^4–6^. Crops are grown seasonally and after harvesting, grains are stored for short or long periods as food reserves and as seeds for next season.

Postharvest loss is a major challenge for food security in the developing world, which can occur across the food supply chain from the harvesting of crops until their consumption^7^. Postharvest loss is caused by spoilage microorganisms, global warming, and insects, which are characterized by loss of weight, quality, nutrition, seed viability, and commercial value^8,9^. Insects are the primary cause of maize grain loss^10–12^. The maize weevil (*Sitophilus zeamais* [Motschulsky]) and larger grain borer (*Prostephanus truncatus* Horn) are the major pests of maize. Approximately 23% losses were observed in maize grains stored for six months, mainly due to infestation of maize weevils in Benin and 12–44% in the western highlands of Cameroon^12–14^.

In Ethiopia, the most important insect pests that cause damage to maize in the field and storage are lepidopteran stalk borers and coleopteran weevils^15^. In addition, Abraham^16^ reported more than 37 species of arthropod pests are associated with maize grain storage in Ethiopia The average grain loss due to storage insect pests is estimated to be 10–30%^16^. It has been reported that *S. zeamais* can cause heavy infestation of maize and sorghum grains stored in traditional storage facilities, resulting in weight losses of up to 41–80%^17^. Therefore, protection against plant pests is an important issue for agricultural communities. In Compared with boosting crop production to fulfill food demand, strategies to reduce postharvest losses require relatively moderate investment and can yield substantial returns.

There are different methods of combating postharvest losses of maize. Although there many chemicals are available in market for crop protection or prevent losses, the biopesticides like botanical pesticides, entomopathogenic microorganisms and synthetic hormone analogues are used as alternative to harmful synthetic chemical pesticides. Medicinal plants have the ability to synthesize a variety of secondary metabolites or bioactive compounds, such as flavonoids, alkaloids, phenolic compounds, and essential oils (alfa-pinene, beta-pinene, alpha-phellandrene, ocimene, borneol, germacrene-B, and gama-cadinine), which are responsible for insecticides^18^. Bioactive compounds have a variety of effects, including repelling, oviposition or feeding, developmental disturbances, and acute mortality of insect pests^19^.

The secondary metabolites of plants are phytotoxic to some degree, and most are used as botanical pesticides in both crude and commercial formulations against several pests without affecting any of their natural enemies^20^. The uses of botanical insecticides has certain advantages, such as lack of persistence in the environment, low risk to non-target organisms, ecofriendly to the environment, and relatively nontoxic to mammals^21,22^.

Some maize producers use chemicals that are not ecofriendly for storage purposes. Others may use medicinal plants that have insecticidal properties, but unfortunately, only a few of them have been properly evaluated^23^. In most developing countries, such as Ethiopian, protection of maize from postharvest loss is a crucial problem that leads to a huge loss of the product. The test plants in the current study have medicinal background and also used against some pests. Essential oil of *V. amygdalina* (0.3%) was able to protect maize from the maize weevil *S. zeamais* by reducing the number of weevil progeny production and by evoking a high repellant action against weevil without damaging the grain. In addition, *V. amygdalina* have been used to control cowpea bruchid, fungal disease in cowpea and vegetable pests^24^. In some parts of Kenya, *M. lanceolata* is traditionally used for the treatment of helminthes and bacterial infections^25^. In addition, the efficacy of leaf of *M. lanceolata and* other botanicals against maize weevil was studied by Sori^26^ and the result indicates *M. lanceolata* reduced the emergence of new progeny from 80 to 40%. *C. macrostachyus* is used as insecticidal and insect repellent^27^. Papaya leaves have been used to overcome insect pests^28^. Traditionally, the leaves and powdered roots of *C. aurea* is used for the treatment of syphilis, malaria, rabies, diabetes, lung TB, hypertension, diarrhoea, leishmaniasis, elephantiasis, fungal diseases, different swellings, stomach-ache, abscesses, bowel, bladder disorders, to induce uterine contractions^29^. In some parts of Africa and in the Philippines, the burning of fresh leaves of *C. anisata* is utilized to repel mosquitoes^30^. Hence, the objective of this study was to evaluate the efficacy of locally available botanical extracts against the maize weevil.

## Materials and methods

### Rearing of test insect

The study was conducted at Dambi Dollo University, Ethiopia, in a zoological science laboratory from August 2022 to October 2022. The test insects were cultured under laboratory conditions at 23 ± 3 °C and 64.8 ± 9% RH. Limmu variety maize seeds were purchased from the Dambi Dollo University Research Center and disinfected in an oven at 40 °C for 4 h from any prior infestation before use as a substrate for insect rearing^31^. The grains were used as food substrates for the test insects. Fifty test insects were placed in 16 plastic jars of 300 ml capacity containing 200 g of seeds. The jars were covered with nylon mesh to allow ventilation and placed with rubber bands to prevent the escape of weevils and to protect the interference of other insects into the jars. The setup was replicated four times. The parent maize weevils were removed by sieving after two weeks of oviposition, and the maize grains were kept under laboratory conditions until the emergence of the F1 progeny.

### Test plant collection and preparation

Before plant parts collection, since the plants have the traditional medicinal background, the researchers have obtained permission from the gardener, Research and Community Service office and herbarium of Biology Department, Dambi Dollo University. Then, the researchers carefully collected the plants parts following institutional, national and international plant collection guidelines and legislations. The plant parts were collected, pressed, dried, mounted and identified by using Flora of Ethiopia and Eritrea^32^ and by comparing with authentic specimens found in the herbarium of Dambi Dollo University. After identification, the specimens were kept in the Herbarium of Dambi Dollo University.

The collected plant materials (Table 1) were kept in the laboratory room and allowed to air dry for two weeks. Both leaves and seeds were grounded using electric grinder (NIMA, NM-8300, Japan) and sieve to prepare a fine powder following the procedure described by Araya and Emana^33^. The uniform finely divided powder was weighed and then the powder sample was kept and packed for extraction purposes.

**Table 1:** Names of collected test plants.

Local name	Scientific name	Family name	Voucher No	Part used	Area of collection
Abbayyii	*Maesa lanceolata* Forssk	Primulaceae	GT016	Seed and leaf	Maxa and Arere
Bakkanniisa	*Croton macrostachyus* Hochst. ex Del	Euphorbiaceae	GT052	Seed and Leaf	D/Dollo town
Pappayyaa	*Carica papaya* Linn	Caricaceae	GT06	Leaf	D/Dollo town
Ceekaa	*Calpurnia aurea* (Ait.) Benth	Fabaceae	GT048	Seed and Leaf	D/Dollo town
Ulmaayii	*Clausena anisata* Hook.f. ex Benth	Rutaceae	GT059	Leaf	Mexi
Eebicha	*Vernonia amygdalina* Del	Asteraceae	GT028	Leaf	DaDU campus

### Solvent extract of plant materials

Powdered plant parts were successfully extracted using a maceration method. The powder (200 g) was weighed using a digital balance. The samples were kept in 2000 ml Erlenmeyer flasks and extracted using solvents such as petroleum ether, chloroform, acetone, methanol, and distilled water. Six hundred milliliter of Petroleum ether (600 mL) was added to a 2000 ml conical flask, and the mixture was shaken well and soaked (macerated) for 72 h at room temperature with gentle shaking twice daily. The petroleum ether extract was filtered through Whatman No.1 filter paper. The marc obtained after filtration was further extracted by the same procedure using chloroform. The same procedure was followed according to polarity using acetone, methanol, and distilled water. The supernatant was collected and the solvent was evaporated at 44–45 °C. The crude extract was placed in a refrigerator at 4 °C in a beaker and covered with aluminum foil until required for insecticidal activity following Bogalech et al.^34^ procedures with slight modifications.

#### Phytochemical study

Phytochemical studies were conducted to identify phytochemicals in the petroleum ether, Chloroform, Acetone, Methanol, and water extracts of the test plants, and the phytochemicals were detected using color tests.

1. Test for alkaloids: Dragendorff’s test: 2 ml of extract was placed into a test tube and acidified with a few drops of dilute hydrochloric acid. Then 1 ml of potassium bismuth iodide solution (Dragendorff’s reagent) was added and the mixture was shaken. An orange-red precipitate indicates the presence of alkaloids^35^.
2. Test for Tannins: To 1 ml of the extract, 2 ml of 5% ferric chloride was added. The formation of dark blue or greenish-black indicates the presence of tannins^36^.
3. To test for saponins, 2 ml distilled water was added to 2 ml of extract and shaken in a test tube. The appearance of the foam indicates the presence of saponins^36,37^.
4. Test for Flavonoids: To 2 ml of the extract, 1 ml of 2N sodium hydroxide was added. The yellow color indicates the presence of flavonoids^36,37^.
5. Test for Glycosides: Method 1. One milliliter of each extract was then collected. Subsequently, a few drops of glacial acetic acid, ferric chloride and 3–4 drops of sulfuric acid were added. The blue-green color indicates the presence of glycosides^37^.
  Method 2. To 2 ml of the extract, 3 ml of chloroform and 10% ammonia solution were added. The pink color indicates the presence of glycosides^36^.
6. Test for Quinones: To 1 ml of extract, 1 ml of concentrated sulfuric acid was added. The formation of a red color indicates the presence of quinones^36^.
7. Test for phenols: To 1 ml of the extract, 2 ml of distilled water followed by a few drops of 10% ferric chloride was added, and the formation of blue or green color indicated the presence of phenols^36^.
8. Test for Terpenoids: To extract the extract (0.5 ml), chloroform (2 ml of chloroform and concentrated sulfuric acid were added. The formation of a red-brown color at the interface indicates the presence of terpenoids^36^.
9. Test for Cardiac Glycosides: To 0.5 ml of the extract, 2 ml of glacial acetic acid and a few drops of ferric chloride were added. The formation of a brown ring at the interface indicated the presence of cardiac glycosides^36^.
10. Test for Coumarins: To 1 ml of the extract, 1 ml of 10% sodium hydroxide was added, and the yellow color indicated the presence of coumarins^36^.
11. Test for Steroids: An equal volume of chloroform followed by a few drops of concentrated sulfuric acid was added to 1 ml of extract, and the appearance of a brown ring indicated the presence of steroids^36^.

### Application of extracts

Different concentrations of the extracts (filtrate) were applied to maize seeds at the rate of 10 ml, 15 ml and 20 ml per 100 g that were placed in glass jar of 0.5 L contained disinfected maize seeds and mixed by shaking following the method of Bekele^31^. Then, 1 ml of distilled water was added to each treated filter paper to moisten it and as the carrier of the active plant material to the insect body. Other glass jars were also treated with three concentrations of the solvent as a negative control. Untreated maize seeds were used as positive controls. After the treatment, 30 maize weevils were introduced into the treated and control glass jars. The experiment was a completely randomized design (CRD) and replicated three times. The mortality of the adult insects was counted at 2, 7, 14, and 28 days after treatment.

### Mortality assessment

The percentage insect mortality was calculated using the following equatoin^38^.

$${\text{Mortality}}\left(\text{\%}\right)=\frac{\text{Number of dead insects}}{Total \;number \;of \;insects}\times 100$$

### F1 progeny assessment bioassay

The jars were kept for an additional 14 days of oviposition time after the mortality assessment.

Then, the assessment was started by removing all dead and alive adult weevils from the maize by sieving. The treated and control grains were kept until the emergence of the F1 progeny. The number of F1 progeny of the maize weevil was then counted to avoid overlapping generations. The counting period of F1 was established so as to avoid an overlap of population generations: the number of F1 progeny produced was recorded daily for 14 days from the time of first adult emergence. i.e. from 6th week. The experiment was ended by 8 weeks after introducing the parental generation.

Following the methods used by Araya and Emana^33^, the formula of percentage reduction in adult emergence or inhibition rate (% IR) was used to determine which treatment inhibited the emergence of the F1 progeny.

$$\left( {\% {\text{IR}}} \right) \, = \frac{{{\text{Total F1 progeny in control }}{-}{\text{ Total F1 progeny in treatment}}}}{{\text{Total F1 progeny in control}}} \times { 1}00$$

where, IR: Inhibition Rate

### Grain damage and weight loss assessment

The percentage of weight loss of maize grains due to insect pests was calculated using the gravimetric or count and weight method^39^ as follows:

$$\text{\%Weight Loss}=\frac{\left({\text{Wu}}*{\text{Nd}}\right)-({\text{Wd}}*{\text{NU}})}{{\text{Wu}}\left({\text{Nd}}+{\text{NU}}\right)}{\text{x}}100$$

where: Wu = weight of undamaged grain Nu = Number of undamaged grain, Wd = Weight of damaged grain Nd = Number of damaged grain

The percentage of insect-damaged seeds was calculated according to Wambugu et al.^40^.

$$\text{Insect damaged grain}\left(\text{\%}\right)=\frac{\text{Number of insect damaged grain}}{Total \;number \;of \;grains}\times 100$$

### Germination test

Twenty seeds from each treatment and control group were placed separately in Petri dishes containing moistened filter paper with 10 ml distilled water. Each treatment was replicated four times (20 seeds per Petri dish) and incubated at room temperature for 4–7 days. The number of emerged seedlings from each petri dish was counted and recorded. The percent germination was computed using Dubale et al.^41^:

$$\text{Germination }(\text{\%})=\frac{\text{Number of germinated seed}}{\text{Number of seed planted}}\times 100$$

### Data analysis

Analysis of variance (ANOVA) was performed using IBM SPSS (version 25) to determine the effect of the treatments on % mortality, number of F1 progeny reduction, % weight loss, grain damage, and effect of the treatments on seed germination. Means were compared using Tukey’s honestly significant difference test at the 5% significance level.

## Results

### Phytochemical screening

The different phytochemicals were qualitatively characterized in terms of tannins, saponins, alkaloids, flavonoids, glycosides, quinones, phenols, terpenoids, cardiac glycosides, coumarins, and steroid (Table 2).

**Table 2:** Phytochemical screening of some botanical extracts.

Plant name (parts)	Solvent used for extraction	Phytochemicals in the crude extract										
		Tannin	Saponin	Alkaloid	Flavonoid	Glycoside	Quinone	Phenol	Terpenoid	Cardiac glycoside	Coumarin	Steroid
*C. macrostachyus* (seed)	Petroleum ether	+	−	+	+	+	+	−	+	−	+	+
	Chloroform	−	−	+	+	+	+	+	+	+	−	+
	Acetone	−	−	+	+	−	+	+	+	+	+	+
	Methanol	−	+	+	+	+	−	−	−	−	+	+
	Water	−	+	+	−	−	+	−	+	+	+	+
*C. macrostachyus* (leaf)	Petroleum ether	+	+	+	+	+	−	−	−	−	+	−
	Chloroform	+	+	+	−	+	−	−	−	−	−	+
	Acetone	+	+	+	+	+	−	+	−	−	+	+
	Methanol	+	+	+	−	−	−	−	−	−	+	−
	Water	+	+	−	−	−	+	−	+	+	−	+
*M. lanceolata* (leaf)	Petroleum ether	−	+	+	−	+	−	−	−	+	+	+
	Chloroform	−	+	+	−	+	−	−	−	+	−	+
	Acetone	+	+	+	+	−	−	+	−	−	−	+
	Methanol	+	+	+	−	−	+	−	−	−	−	+
	Water	+	+	+	−	−	−	+	+	+	−	+
*M. lanceolata* (seed)	Petroleum ether	−	−	+	−	+	−	−	+	−	+	+
	Chloroform	−	−	+	−	+	−	−	−	+	−	−
	Acetone	+	−	+	+	−	−	+	−	−	+	+
	Methanol	+	+	+	+	−	+	−	−	−	+	−
	Water	−	−	+	+	−	+	−	+	+	+	+
*V. amygdalina* (leaf)	Petroleum ether	+	−	+	+	+	−	−	−	+	+	−
	Chloroform	+	+	+	+	−	−	−	−	+	−	+
	Acetone	+	+	+	+	−	−	+	−	−	+	+
	Methanol	+	+	+	−	−	+	+	−	−	+	+
	Water	+	+	+	−	−	+	+	+	−	−	+
*C. papaya* (leaf)	Petroleum ether	+	−	+	+	+	−	−	−	−	+	+
	Chloroform	+	−	+	+	+	−	−	−	+	−	+
	Acetone	+	+	−	+	−	−	+	−	−	−	+
	Methanol	+	+	+	−	−	+	+	−	−	−	+
	Water	+	+	+	−	−	+	+	+	−	−	+
*C. anisata* (leaf)	Petroleum ether	+	+	+	+	+	−	−	−	−	−	+
	Chloroform	−	+	+	+	+	−	−	−	+	−	+
	Acetone	+	+	−	+	−	−	+	−	−	−	+
	Methanol	+	+	+	+	+	−	+	−	−	+	+
	Water	+	+	+	+	−	+	+	+	+	+	+
*C. aurea* (seed)	Petroleum ether	−	−	+	+	+	−	−	+	−	+	−
	Chloroform	−	+	+	+	+	−	−	−	+	−	+
	Acetone	−	+	+	+	−	+	+	−	−	+	+
	Methanol	+	+	+	+	−	−	+	−	−	+	+
	Water	−	+	+	+	−	+	−	+	+	+	+
*C. aurea* (leaf)	Petroleum ether	+	−	+	+	+	−	−	−	+	+	−
	Chloroform	−	+	+	+	+	−	−	−	+	−	+
	Acetone	+	+	+	+	−	−	+	−	−	+	+
	Methanol	+	+	+	−	−	+	+	−	−	−	+
	Water	−	+	+	+	−	+	−	+	+	+	+

### Effect of botanical extracts on Sitophilus zeamais mortality and F1 progeny emergence

The results in Table 3 show the effectiveness of nine botanical extracts with five solvent extractions (petroleum ether, chloroform, acetone, methanol, and distilled water) at different application rates (i.e., 10, 15, and 20 ml). The results revealed that all the treatments with different extracts at different rates of application showed significant differences (p < 0.05) in the cumulative mean percent mortality of the maize weevil, which was higher than the percent mortality (2.33%) recorded from the untreated control. After 10 ml treatment, the maximum cumulative percent mortality (95.55% and 95.32%) were recorded with the seed extracts of *C. macrostachyus* and *M. lanceolata* followed by leaf extract of *C. anisata, C. aurea* seed and *C. papaya* leaf extract. The minimum cumulative mean percent mortality (88.43%) was recorded with the leaf extract of *C. macrostachyus* which was higher than the mean percent mortality (2.33%) with the untreated control. After 15 ml treatment, the maximum cumulative mean percent mortality (96.89%) was recorded with the leaf extract of *M. lanceolata*. The minimum cumulative mean percent mortality (90.44%) was recorded with the leaf extract of *C. macrostachyus*. After 20 ml treatment, the maximum cumulative mean percent mortality (98.68%) was recorded with the leaf extract of *C. papaya*. The minimum cumulative mean percent mortality (94.44%) was recorded for the *C. macrostachyus* leaf extract.

**Table 3:** Efficacy of different botanical extracts on mean percentage mortality of maize weevil, *S. zeamais.*

Treatment	Cumulative mean percent mortality by different extracts concentrations		
	10 ml (mean ± SD)	15 ml (mean ± SD)	20 ml (mean ± SD)
*M. lanceolata* seed	95.32 ± 3.75^ab^	96.89 ± 2.67^a^	98.02 ± 1.67^a^
*M. lanceolata* leaf	90.23 ± 5.41^abc^	93.99 ± 3.39^ab^	96.22 ± 3.31^ab^
*C. macrostachyus* seed	95.55 ± 3.72^a^	96.44 ± 2.96^a^	98.45 ± 2.13^a^
*C. macrostachyus* leaf	88.43 ± 5.75^c^	90.44 ± 4.33^b^	94.44 ± 4.48^b^
*C. aurea* leaf	91.98 ± 4.69^abc^	94.22 ± 4.08^ab^	96.01 ± 4.74^ab^
*C. aurea* seed	92.45 ± 5.97^abc^	94.65 ± 3.75^a^	97.11 ± 2.79^ab^
*C. papaya* leaf	92.44 ± 4.44^abc^	95.11 ± 3.31^a^	98.68 ± 1.67^a^
*V. amygdalina* leaf	89.99 ± 3.98^bc^	93.32 ± 3.34^ab^	97.35 ± 2.26^ab^
*C. anisata* leaf	92.89 ± 4.15^abc^	95.55 ± 3.01^a^	98.01 ± 2.46^a^
Untreated control	2.33 ± 1.15^d^		

Note: Means followed by different letters down the column are significantly different.

The results of the F1 emergence are presented in Table 4. There was no significant difference (p > 0.005) in all treatments, except that the inhibition rate was 0% in the untreated control. Complete inhibition (100%) of the F1 progeny emergence was recorded with the leaf extract of *C. aurea* at all application rate*s*. The seed extract of *C. macrostachyus* and leaf extract of *V. amygdalina* i revealed 100% inhibition of the F1 emergence at 15 and 20 ml application rate.

**Table 4:** Different botanical treatments to maize grains and their effect on F1 Progeny emergence of *S. zeamais.*

Treatment	Cumulative mean percent mortality by different extracts concentrations		
	10 ml (mean ± SD)	15 ml (mean ± SD)	20 ml (mean ± SD)
*M. lanceolata* seed	1.99 ± 0.02^a^	1.99 ± 0.01^a^	2.00 ± 0.00^a^
*M. lanceolata* leaf	1.98 ± 0.03^ab^	1.99 ± 0.02^a^	1.99 ± 0.01^a^
*C. macrostachyus* seed	1.99 ± 0.03^a^	2.00 ± 0.00^a^	2.00 ± 0.00^a^
*C. macrostachyus* leaf	1.96 ± 0.05^b^	1.97 ± 0.01^b^	1.98 ± 0.03^b^
*C. aurea* leaf	1.99 ± 0.02^a^	1.99 ± 0.01^a^	2.00 ± 0.00^a^
*C. aurea* seed	1.99 ± 0.02^a^	1.99 ± 0.02^a^	2.00 ± 0.00^a^
*C. papaya* leaf	1.99 ± 0.02^a^	2.00 ± 0.00^a^	2.00 ± 0.00^a^
*V. amygdalina* leaf	1.99 ± 0.02^a^	2.00 ± 0.00^a^	2.00 ± 0.00^a^
*C. anisata* leaf	2.00 ± 0.00^a^	2.00 ± 0.00^a^	2.00 ± 0.00^a^
Untreated control	0.00 ± 0.00^c^		

Note: Means followed by the same letters down the column are not significantly different.

### Effect of direct application of botanical extracts on maize grain damage, weight loss and seed germination

The results of the maize grain weight loss due to maize weevils are presented in Table 5. There was a significant difference (p < 0.05) among all treatments at all application dosages. The highest maize grain weight loss was recorded from the leaf extract of *C. macrostachyus* (5.46%) at a 10 ml application rate; the lowest maize grain weight loss due to maize weevil was recorded from the leaf extract of *C. anisata* with methanol extraction (2.63%) at the same application dosage. The highest grain weight loss was recorded from the leaf extract of *C. macrostachyus* (4.65% and 3.81%) and the lowest was recorded in the leaf extract of *C. anisata* (1.44% and 0.91%) respectively, at 15 ml and 20 ml application rates.

**Table 5:** Application of different botanical extracts to maize grains and their effect on grain weight loss.

Treatment	Cumulative mean percent mortality by different extracts concentrations		
	10 ml (mean ± SD)	15 ml (mean ± SD)	20 ml (mean ± SD)
*M. lanceolata* seed	5.23 ± 0.69^a^	3.81 ± 0.83^ab^	3.20 ± 0.98^ab^
*M. lanceolata* leaf	4.83 ± 0.99^ab^	3.62 ± 0.94^ab^	2.99 ± 1.29^ab^
*C. macrostachyus* seed	4.55 ± 0.78^ab^	4.33 ± 1.02^a^	3.67 ± 1.21^a^
*C. macrostachyus* leaf	5.46 ± 1.06^a^	4.65 ± 0.98^a^	3.81 ± 0.95^a^
*C. aurea* leaf	3.16 ± 1.12^ cd^	2.82 ± 1.03^bcd^	1.88 ± 0.81^ cd^
*C. aurea* seed	4.57 ± 0.90^ab^	3.36 ± 0.81^abc^	2.35 ± 0.57^bc^
*C. papaya* leaf	3.27 ± 0.91^ cd^	2.11 ± 0.69^de^	1.21 ± 0.73^d^
*V. amygdalina* leaf	3.93 ± 0.82^bc^	2.61 ± 0.93^ cd^	1.71 ± 0.78^ cd^
*C. anisata* leaf	2.63 ± 1.04^d^	1.44 ± 1.04^e^	0.91 ± 0.68^d^
Untreated control	67.37 ± 8.73^a^		

Note: Means followed by different letters down the column are significantly different.

The highest grain damage (67.37%) was observed in the untreated control. Table 6 shows the maize grain damage by maize weevils during the experimental period. There were significant differences (p < 0.05) among all treatments at all dosages. The highest grain damage was recorded from the leaf extract of *C. macrostachyus* (11.63%), followed at a 10 ml rate of application. The lowest content (5.67%) was recorded in the leaf extract of *C. anisata*. At the application rate of 15 mL, 10.41%, was recorded from the leaf extract of *C. macrostachyus*. The lowest content (4.32%) was recorded in the leaf extract of *C. anisata*. The highest (9.47%) and lowest grain damage were recorded from the leaf extract of *C. anisata* (3.25%), respectively, at a 20 ml rate of treatment application. The highest grain damage (90.97%) was observed in the untreated control.

**Table 6:** Application of different botanical extracts to maize grains and their effect on grain damage.

Treatment	Cumulative mean percent mortality by different extracts concentrations		
	10 ml (mean ± SD)	15 ml (mean ± SD)	20 ml (mean ± SD)
*M. lanceolata* seed	7.81 ± 1.71^c^	6.91 ± 1.62^ cd^	5.87 ± 1.89^bcd^
*M. lanceolata* leaf	8.37 ± 1.47^bc^	7.67 ± 1.74^bc^	6.61 ± 1.68^ab^
*C. macrostachyus* seed	9.91 ± 1.68^ab^	8.95 ± 1.96^ab^	7.61 ± 1.44^ab^
*C. macrostachyus* leaf	11.63 ± 1.70^a^	10.41 ± 1.15^a^	9.47 ± 1.28^a^
*C. aurea* leaf	7.48 ± 1.57^ cd^	5.91 ± 0.92^de^	4.83 ± 0.93^de^
*C. aurea* seed	8.87 ± 1.59^bc^	7.51 ± 1.25^bcd^	5.75 ± 0.99^bcd^
*C. papaya* leaf	7.43 ± 1.59^ cd^	5.97 ± 1.47^de^	4.97 ± 1.16^ cd^
*V. amygdalina* leaf	8.46 ± 1.57^bc^	7.45 ± 1.58^bcd^	6.47 ± 1.54^abc^
*C. anisata* leaf	5.67 ± 1.62^d^	4.32 ± 1.23^e^	3.25 ± 1.29^e^
Untreated control	90.97 ± 2.53^a^		

Note: Means followed by different letters down the column are significantly different.

Table 7 presents the results of maize seed germination under different application rates of the different botanical extracts. There were significant differences (p < 0.05) among the treatments. Seed germination recorded in the untreated control was only 22.5%. Leaf extract of *C. anisata* showed 92.5% seed germination at 10 ml application dosage. The highest maize seed germination (94.75%) was recorded in the leaf extract of *C. anisata* while the lowest (78.75%) was obtained from leaf extract of *C. macrostachyus* at a treatment application rate of 15 ml. At a 20 ml rate of treatment application, 97% seed germination was recorded with leaf extract application of *C. anisata*. At the same application rate, leaf extracts of *C. macrostachyus* showed 85.5% maize seed germination.

**Table 7:** Effect of botanical extracts treatment on maize seed germination.

Treatment	Cumulative mean percent mortality by different extracts concentrations		
	10 ml (mean ± SD)	15 ml (mean ± SD)	20 ml (mean ± SD)
*M. lanceolata* seed	83 ± 5.71^bc^	88.75 ± 3.58^bc^	92.5 ± 2.56^abc^
*M. lanceolata* leaf	81.25 ± 6.86^bc^	86 ± 6.19^c^	90 ± 5.38^bcd^
*C. macrostachyus* seed	81 ± 7.18^bcd^	86 ± 5.28^c^	90.75 ± 4.94^bc^
*C. macrostachyus* leaf	74.5 ± 9.72^d^	78.75 ± 9.98^d^	85.5 ± 7.59^d^
*C. aurea* leaf	86.75 ± 6.34^ab^	92.25 ± 4.72^ab^	94.5 ± 4.26^ab^
*C. aurea* seed	79 ± 3.84^ cd^	85.75 ± 5.68^c^	88.25 ± 5.19^ cd^
*C. papaya* leaf	87.5 ± 5.96^ab^	90.75 ± 4.37^abc^	94.25 ± 4.06^ab^
*V. amygdalina* leaf	86.75 ± 6.93^ab^	91.5 ± 5.64^abc^	94 ± 4.47^ab^
*C. anisata* leaf	92.5 ± 5.73^a^	94.75 ± 4.72^a^	97 ± 2.99^a^
Untreated control	22.5 ± 10.5^e^		

Note: Means followed by different letters down the column are significantly different.

## Discussion

Extracted secondary metabolites from different plant species with polarity of different solvents caused mortality of maize weevils, which is in agreement with the work of other researchers. Mbata et al.^42^ reported that alkaloids can affect nerve transmission in insects by disturbing the cell membrane and cytoskeletal structure, causing the collapse and leakage of cells. Ma et al.^43^ also reported that alkaloids have a variety of biological activities, such as poisoning, antifeeding, and inhibition of insect growth and development. Kazemi^44^ reported that the secondary metabolite saponin acts as a detergent that disrupts the cell membrane, causes cell death, and ultimately kills insect pests. In addition, a report by the European Medicine Agency^45^ indicated that the insecticidal activity of saponins is mediated via an interaction with cholesterol, which disrupts the synthesis of steroids from ecdysis. According to Salminen and Karonen^46^, phenols have antifeedant, toxic, and regulatory activities that affect insect physiological processes or repel phytophagous insects.

Tlak Gajger and Dar^47^ reported that tannins cause toxicity in insects. Pizzolitto et al.^48^ reported the biological activity of terpenoids with different structural groups against stored grain pests, with ketones being the most biologically active group. Studies have shown that flavonoids such as rotenone are effective insect repellents. Huang et al.^49^ in their review article reported the antifeedant activity of rotenone against *S. granarius* and *T. confusum* adults, and found that it showed the strongest deterrent effect and was the best antifeedant tested. Akhtar et al.^50^ tested the insecticidal activities of quinones, and their results indicated that quinones are acutely toxic against two-spotted spider mites and three aphid species. Al-Rajhy et al.^51^ reported that cardiac glycosides have promising pesticidal effects. Mukandiwa et al.^52^ reported that coumarin extracted from *C. anisata* leaves inhibited feed intake in the first and second instars of blowfly larvae and resulted in significantly lower mass pupae. Zhang et al.^53^ also reported that coumarins have feed deterrent activities against *T. castaneum* adults.

A recent study conducted by Verma et al.^54^ to isolate compounds with pesticidal properties from *G. sessilifora* Sims revealed that the identified compounds were good protectants against pests. Zain et al.^55^ also identified phytochemical compounds, such as flavonoids, saponins, tannins, steroids, phenolics, and alkaloids, and explained the potential insecticidal activity of these compounds. Boualam et al.^56^ studied the phytochemical composition of two botanicals using different solvents (petroleum ether (PE), dichloromethane (DCM), dichloromethane/methanol (80/20), and methanol) and identified polyphenols, alkaloids, flavonoids, tannins, sterols, terpenoids, and saponosides.

In the current study, all plant extracts showed significant effects against maize weevils at all the tested concentrations (10, 15, and 20 ml) after 28 days of exposure. This result is in agreement with the work of Islam et al.^57^, who reported that the mean mortality in maize weevils increased with an increase in concentration. They also indicated that different plant extracts showed significant effects on maize weevils at 5, 10, and 15% concentrations.

No adult progeny emerged from maize seeds treated with *C. anisata* leaf extract at any of the applied dosages. The percentage inhibition rate of the untreated control was 0%. This result is consistent with the result reported by Ileke^58^, in which no adult emergence was observed in wheat grains treated with *C. frutescens* and *A. occidentale* extracts at all tested concentrations, and adult emergence was significantly higher (p < 0.05) in the control than in the treated wheat grains.

In the present study, the mean percentage of grain damage and grain weight loss was reduced with an increased treatment dosage. All botanical extracts had different effects on the protection of grain damage. Gariba et al.^59^ indicated that the mean percentage weight loss and seed damage were lower at higher concentrations than at lower concentrations. Moreover, Parwada et al.^60^ reported that botanicals reduce the occurrence of a weevil attack if the concentration is increased.

All botanical extracts supported maize seed germination, which was significantly higher than germination as shown by the untreated control, which is in line with the report of Abou El-Nour and Ewais^61^, who showed that moringa leaf extract enhances pepper seed germination percentage. Gariba et al.^59^ revealed that seeds treated with botanical extracts had significantly higher germination rates than the values obtained in untreated seeds. Their results indicated that the seeds were viable and had a good germination percentage.

## Conclusion

The presence of bioactive compounds in different botanical extracts has shown that the selected plants are promising grain protectants. At all levels (10, 15, and 20 ml) of treatment application, *M. lanceolata* seed extract, *C. macrostachyus* seed extract and *C. anisata* showed percentage mortality ranged from 95.32 to 98.02% after 28 days of exposure. Therefore, the extract of these plants have a promising effect on maize weevil mortality for the protection of stored maize. *C. anisata* showed a 100% inhibition rate of F1 progeny emergence at all application rates.

The highest grain weight loss (5.25–5.46%) was recorded in the seed extract of *M. lanceolata* and leaf extract of *C. macrostachyus* at a 10 ml application rate. The lowest weight losses, 1.44% and 0.91%, were calculated from the applied *C. anisata* leaf extract at 15 and 20 ml, respectively. With increased dosage, this plant extracts reduced grain weight loss. *C. anisata* showed the lowest maize grain damage for all treatment applications. Relative to other botanical extracts, *C. macrostachyus* leaf extract showed the lowest seed germination percentage. Quantifying bioactives, testing the insecticidal activity of low-dose (< 10 ml) extracts, and separating and purifying to obtain effective chemical components from these botanicals are required in future research.

## Data Availability

The data used to support the findings of this study are included within the article.
